# A Virtual Reality-Based Self-Help Intervention for Dealing with the Psychological Distress Associated with the COVID-19 Lockdown: An Effectiveness Study with a Two-Week Follow-Up

**DOI:** 10.3390/ijerph18158188

**Published:** 2021-08-02

**Authors:** Giuseppe Riva, Luca Bernardelli, Gianluca Castelnuovo, Daniele Di Lernia, Cosimo Tuena, Alex Clementi, Elisa Pedroli, Clelia Malighetti, Francesca Sforza, Brenda K. Wiederhold, Silvia Serino

**Affiliations:** 1 Humane Technology Lab., Università Cattolica del Sacro Cuore, 20123 Milan, Italy; giuseppe.riva@unicatt.it; 2 Applied Technology for Neuro-Psychology Lab., IRCCS Istituto Auxologico Italiano, 20149 Milan, Italy; cosimo.tuena@unicatt.it (C.T.); e.pedroli@auxologico.it (E.P.); 3 Become-Hub, 20123 Milan, Italy; luca.bernardelli@become-hub.com (L.B.); francesca.sforza@become-hub.com (F.S.); 4 Department of Psychology, Università Cattolica del Sacro Cuore, 20123 Milan, Italy; gianluca.castelnuovo@unicatt.it (G.C.); daniele.dilernia@unicatt.it (D.D.L.); alex.clementi01@icatt.it (A.C.); clelia.malighetti@unicatt.it (C.M.); 5 Faculty of Psychology, University of eCampus, 22060 Novedrate, Italy; 6 Virtual Reality Medical Center, La Jolla, CA 92037, USA; b@vrphobia.eu; 7 Virtual Reality Medical Institute, 1200 Brussels, Belgium

**Keywords:** psychological stress, virtual reality, COVID-19, well-being, digital technology, self-help technology

## Abstract

The aim of this study is to investigate the effectiveness of a novel self-administered at-home daily virtual reality (VR)-based intervention (COVID Feel Good) for reducing the psychological burden experienced during the COVID-19 lockdown in Italy. A total of 40 individuals who had experienced at least two months of strict social distancing measures followed COVID Feel Good between June and July 2020 for one week. Primary outcome measures were depression, anxiety, and stress symptoms, perceived stress levels, and hopelessness. Secondary outcomes were the experienced social connectedness and the level of fear experienced during the COVID-19 pandemic. Linear mixed-effects models were fitted to evaluate the effectiveness of the intervention. Additionally, we also performed a clinical change analysis on primary outcome measures. As concerning primary outcome measures, participants exhibited improvements from baseline to post-intervention for depression levels, stress levels, general distress, and perceived stress (all *p* < 0.05) but not for the perceived hopelessness (*p* = 0.110). Results for the secondary outcomes indicated an increase in social connectedness from T0 to T1 (*p* = 0.033) but not a significant reduction in the perceived fear of coronavirus (*p* = 0.412). Among these study variables, these significant improvements were maintained from post-intervention to the 2-week follow-up (*p* > 0.05). Results indicated that the intervention was associated with good clinical outcomes, low-to-no risks for the treatment, and no adverse effects or risks. Globally, evidence suggests a beneficial effect of the proposed protocol and its current availability in 12 different languages makes COVID Feel Good a free choice for helping individuals worldwide to cope with the psychological distress associated with the COVID-19 crisis, although large scale trials are needed to evaluate its efficacy.

## 1. Introduction

In late 2019, the virus responsible for the severe acute respiratory syndrome coronavirus 2 COVID-19 was first identified in a small Chinese town [1,2] forcing the world to face unprecedented medical, economic, and social challenges. After an uncontrollable diffusion of the disease from China to other countries, the World Health Organisation (WHO) declared the COVID-19 outbreak a ‘Public Health Emergency of International Concern’ on 30 January 2020. The immense spread of the COVID-19 and the consequent health emergency raised by the growing number of reported cases worldwide led the WHO to declare it a ‘pandemic’ on 11 March 2020 [3].

During the first three months of the emergency, Italy was one of the most affected countries [4]. The first cases were confirmed between 21 and 24 February 2020 in two areas in the northern regions of Italy, namely, the Lombardy and Veneto regions. Two ‘red zones’ were quickly introduced to isolate those areas, introducing physical/social distancing measures to slow down the spread of the disease. On 8 March, the Italian government extended these measures to all Lombardy and then to the whole country (the so-called Fase-Uno). Maintaining a social distance of at least 2 m away from other individuals, and limiting social interactions became the first rules to contain the transmission of the virus. Schools and all non-essential businesses were closed, social events were prohibited, and working from home became mandatory unless this was impossible due to the nature of the business or activities. The stay-at-home order (also called lockdown) resulted in a mass isolation measure that confined individuals to their homes except for essential needs [5,6]. These measures were implemented worldwide with varying degrees, aiming to limit the devasting diffusion of this disease, and were partially removed in Italy on 4 May 2020 when the so-called Fase-Due began. These measures globally achieved their goals of containing the dramatic spread of the disease; however, they also lead to negative psychological consequences and degradation of normal social systems [7,8,9,10,11]. Recent reviews indicated that the short-term psychological consequences included post-traumatic and depressive symptoms, high perceived stress, and an increase in anxiety and sleep disorders [6,12,13,14,15]. A study carried out during the first three weeks of the lockdown in Italy indicated that 37% of participants experienced post-traumatic stress, while 17–23% reported significant levels of anxiety, perceived stress, and adjustment disorders [16]. Some authors underlined that such effects can be pervasive and long-lasting, suggesting that the exposure to the psychological stress experienced during the ‘quarantine’ and the consequent social isolation can provoke long-term effects on mental health [17,18]. As reported by Gruber et al. [19], there are three peculiar and unique ways in which the COVID-19 pandemic is a stressor able to adversely affect mental health in the global population. First, the pandemic is accompanied by feelings of uncertainty regarding its duration that resulted in increased and diffuse concerns about its long-term economic and social consequences [20,21,22]. Second, the COVID-19 pandemic is a multidimensional stressor, affecting at the same time multiple spheres of our lives: work, family, and social life. The lockdown period forced families to try to maintain a functional work-family balance meeting the unique demand of the new working-from-home environment merged with homeschooling [23]. In addition, although economic-related stressors are always present, the COVID-19 crisis has rapidly intensified their load in terms of a sudden increase of unemployment and salary cuts that raise huge uncertainty about the future economic stability [24]. Third, social distancing measures adopted worldwide have had enormous costs on the social processes that underlie the very fabric of our society [25,26]. On one hand, these measures can negatively impact mental health in terms of an increase in perceived social isolation, personal distress [8,25,27], and unhealthy behaviours (e.g., excessive substance use; see, for example, [28]). In addition, adhering to stay-at-home orders and adopting social distancing measures drastically reduce the possibility to access important protective factors against stress, such as active participation in meaningful social relationships and interactions [29,30].

Delivering evidence-based psychological interventions for both preventing and treating these mental health disorders is an urgent challenge, considering also that the social distancing measures may limit the adoption of traditional psychological services [7,31]. To facilitate access to psychological services, traditional psychological interventions have been recently reconfigured to online or telehealth formats [31]. The literature suggests the efficacy of telehealth for delivering psychological interventions [32,33,34], and recent studies demonstrated the feasibility of these approaches during the COVID-19 crisis for both health care professionals and the general population [35,36,37,38]. In recent years, there has been a huge increase in the use of technology for the implementation of validated psychological interventions for a wide variety of psychological problems [33,39,40,41]. Digital psychological self-help interventions can be accessed from any location, with an obvious decrease in logistic and geographical barriers to treatment delivery. As a result, they are often more affordable and less time consuming in comparison to traditional interventions, with a subsequent lower impact on the public health service and a significant improvement in self-monitoring processes [42,43,44,45,46,47]. In particular, advanced technologies, such as virtual reality (VR) could play a unique role in times of the COVID-19 pandemic by supporting the delivery of accessible and affordable self-guided therapeutic interventions that offer multiple advantages, considering also high-treatment costs and the limited availability of health professionals [31,48,49,50]. VR occupies a prominent place among all other technologies for delivering self-help protocols for several reasons [40,41,51]. It is traditionally employed as a ‘simulative instrument’ [40] to reproduce ecologically valid and interactive scenarios recreating feared/critical situations (i.e., fear of spiders) while allowing the anxiety to attenuate, or naturalistic situations where one can learn specific skills in a safe, ecological, and interactive environment. At the same time, VR-based protocols allow precise control over the sessions’ delivery, according to both therapeutic strategies and patients’ progress; this aspect is particularly relevant for self-help protocols administered in the absence of a therapist’s guidance. These advantages offered by VR have already been exploited in VR-based self-help treatments for treating phobias and anxiety disorders [42,52,53]. 

The present study tested the effectiveness of COVID Feel Good [54], a novel self-help VR-based protocol aimed at helping individuals to cope with the psychological burden related to the COVID-19 pandemic and restrictive social distancing measures. The protocol consists of watching a 10 min, three hundred sixty–degree (360°) VR video, titled ‘The Secret Garden’, at least once a day for a week. Each day, after the VR session, participants were invited to perform a series of social exercises with targeted goals for each day of the week. 

We hypothesised that the daily VR-based self-help protocol COVID Feel Good would be associated with a reduction in levels of depression, stress, anxiety, general distress, perceived stress, and hopelessness (primary outcome measures); an increase in the perceived interpersonal closeness with the social world; a decrease in fear of COVID-19 (secondary outcome measures) at the end of the intervention, compared with baseline responses and those from the 1-week waiting period. We predicted also that treatment gains would be maintained at 2-weeks follow-up. Additionally, we also evaluated the pre–post changes in perceived levels of relaxation and distress by comparing responses collected each day during the 1-week waiting period with those collected after completing each daily treatment module.

## 2. Materials and Methods

### 2.1. Recruitment and Experimental Design

**Figure 1:**
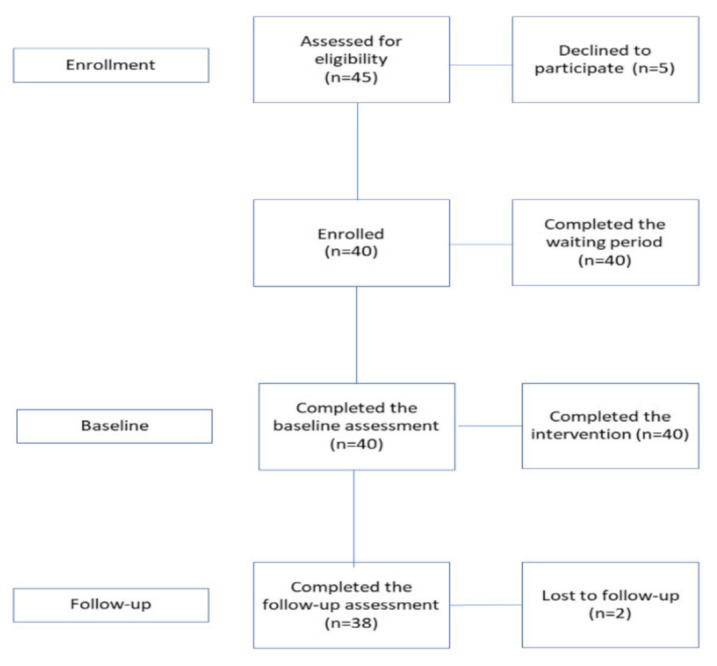
Participant flowchart.

We utilised a before-and-after study design. We compared changes in levels of depression, anxiety, stress, general distress, perceived stress, hopelessness, social connectedness, and fear of COVID-19 between a control waiting period (i.e., 7 days before the start of the intervention—Waiting Period), with changes before the start of the treatment (Day 0—T0) and the end of the treatment period (Day 7—T1). The inclusion of a 2-week follow-up (Day 21—T2) allowed us to additionally evaluate the stability of the treatment effects. State measures of anxiety, perceived stress, mood feelings, and relaxation levels were collected daily in the waiting period (7 days each day before the start of the intervention) and after the completion of each treatment module, from Day 0 to Day 7. From June to July 2020, participants were recruited through the combined use of traditional strategies and advertisements on a social media platform in Italy and completed the intervention. Potential participants were directly contacted by one of the researchers through emails and asked to complete a brief online questionnaire for screening purposes. A total of 45 participants were contacted for screening (Figure 1). The study was approved by the IRCSS Istituto Auxologico Italiano ethical review board in Milan, Italy (2020_06_16_09), and conducted according to the 1964 Declaration of Helsinki. 

Inclusion criteria for participation included the following: (1) being at least 18 years old; (2) living in Italy during the pandemic; (3) being Italian-speaking citizens; (4) having experienced at least two months of strict social distancing measures (including a stay-at-home order) implemented by the Italian National Government (precise information about the measures implemented can be found here [55]); (5) availability and agreement of a partner for conducting the relational component of the treatment; (6) availability of a smartphone with Internet access; (7) normal or corrected-to-normal vision. Exclusion criteria (all self-reported) included the presence of a diagnosis of a major mental disorder, a lack of stereoscopic vision, or a balance/vestibular problem that would disrupt the VR experience. Participants who met the abovementioned eligibility criteria and agreed to participate (N = 40, Figure 1) were provided with information about the study design and asked to sign the informed consent. Table 1 presents the sample sociodemographic characteristics. 

After the inclusion, they received the psychological assessment survey including all the outcome measures to be completed online through a Qualtrics survey and were informed that their intervention would start after a 1-week waiting period. In this initial survey, they were also asked to indicate whether they would have followed the protocol through their smartphone and a basic low-cost cardboard VR headset (‘immersive modality’) or through the YouTube App (‘non-immersive modality’). During the waiting period, participants received each day a Qualtrics survey link to complete the state measures. When the waiting period was over, participants received another Qualtrics survey to complete (the T0 baseline psychological assessment) with instructions to start the protocol and the self-help materials as an email attachment. During the treatment week, each day they received an email with the state assessment battery through a Qualtrics link to be completed at the end of each VR treatment module. At the end of the week (Day 7—T1) and after a 2-week follow-up (Day 21—T2), participants were invited to again complete the assessment survey with the same outcome measures as at baseline.

### 2.2. Treatment Protocol

Participants received six modules consisting of two integrated parts: the first part consists of a 10 min, 360° VR video entitled ‘The Secret Garden’, and the second part includes a series of social exercises, with a specific goal for each day of the week. From a technological viewpoint, 360° videos are spherical videos recorded with omnidirectional cameras to collect images from all around the environment, and they can be captured in natural landscapes or created using 3D computer graphics software (360° VR videos). They can be displayed in immersive (i.e., through a head-mounted display or low-cost cardboard connected with a smartphone) or in a non-immersive way (i.e., for instance, YouTube supports 360° video formats both in its Android app and on its website). During the playback, users can actively move the camera and have a 360° view of the environment. This feature allows them to have multiple views of the same video content interactively, feeling immersed or present in the represented environment and becoming the protagonist of the action that unfolds in front of their eyes. 

**Figure 2:**
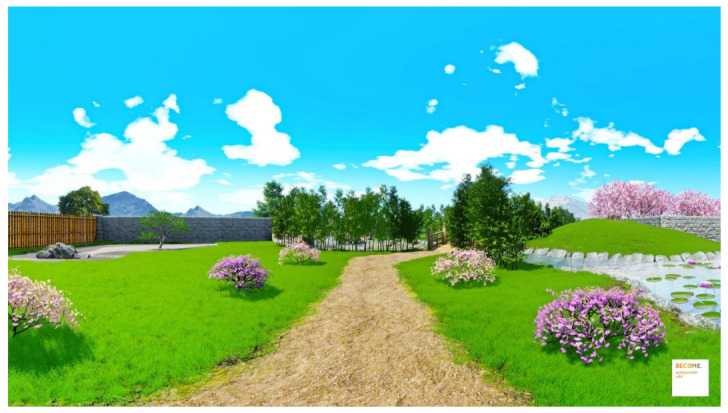
‘The Secret Garden’, a 360° virtual reality scenario where participants are immersed in a naturalistic and safe digital place, far from the stressful situations experienced in routine daily contexts, in which they can learn how to relax and reflect upon their experience following a guided protocol.

The 360° VR video ‘The Secret Garden’ (Figure 2) has been developed through an iterative process involving psychologists, 3D artists, musicians, storytellers, and designers. It has been validated in previous studies by Chirico et al. [56]. It immerses users in a beautiful and relaxing Japanese garden, providing them with the visual (i.e., the flow of water) and auditory (i.e., the sound of running water) elements available in natural environments. The virtual environment has been developed using the software Unreal Engine. To guarantee scalability, the protocol could be accessed through both a participant’s smartphone and a basic low-cost cardboard VR headset (immersive modality) or through the YouTube App (non-immersive modality). The immersive experience is accompanied by a relaxation induction narrative structured following the principles of compassion-focused therapy [57,58]. This relaxation technique aims at deactivating the human threat protection system and activating the soothing system, with a mindset devoted to giving and receiving care, and nurturance. At the end of the VR exposure, participants were invited to perform a series of social tasks related to personal identity and interpersonal relationships. These tasks have been developed following guidelines provided by Winch [59] to cope with emotional experiences that can generate emotional pain, loneliness, rejection, or rumination. The tasks have the following general aims: (1) helping participants to pay attention and recognise their emotional discomfort; (2) supporting participants to reinforce their coping skills; (3) helping participants to monitor themselves and protect self-esteem; (4) support participants in finding a personal meaning even in difficult times. In addition, each activity has a specific objective as follows:

- Day 1: Support to cope with stress, negative intrusive thoughts, worries related to the COVID-19 pandemic;
- Day 2: Increase in self-esteem;
- Day 3: Promotion in the use of autobiographical memories to create a stable representation of themselves over time, as well as increase intimacy and connectedness by sharing personal memories;
- Day 4: Enhanced sense of community;
- Day 5: Promotion in conscious self-regulation and self-organisation of life goals;
- Day 6: Increase in empathy;
- Day 7: Support a long-term positive psychological change.

All the exercises are designed to be experienced with another person (not necessarily physically together) to facilitate a process of critical examination and eventual revision of core assumptions and beliefs related to personal identity, relationships, and goals. The full description of the sessions is provided in [54]. 

### 2.3. Outcome Measures

#### 2.3.1. Primary Outcome Measure

The following self-report questionnaires were included in the psychological assessment survey completed online through Qualtrics and administered at different time intervals to investigate changes in the subjective experience during a control waiting period (7 days before the start of the intervention—Waiting Period), prior to treatment (Day 0—T0) at the end of the treatment period (Day 7—T1), and at 2-week follow-up (Day 21—T2).

- Depression Anxiety Stress Scale (DASS-21) [60,61]: The DASS-21 is the short version of the original self-report questionnaire developed and validated by Lovibond et al. [60] to evaluate depression, anxiety, and stress symptoms. It is composed of 21 items, with 7 items per subscale, namely, (1) DASS-21 Depression, a specific subscale for assessing depressed mood and absence of positive emotions (e.g., ‘I could not seem to experience any positive feeling at all’); (2) DASS-21 Anxiety, a specific subscale for evaluating anxiety feelings and somatic tension and (e.g., ‘I was aware of the dryness of my mouth’); (3) DASS-21 Stress, a specific subscale for evaluating somatic stress, with a focus on difficulty relaxing and irritability; (e.g., ‘I found it hard to wind down’). Participants are asked to score every item on a scale from 0 (‘did not apply to me at all’) to 3 (‘applied to me very much’). Sum scores are computed by adding up the scores on the items for each subscale the three subscales (DASS-21_Depression, DASS-21_Anxiety, DASS-21_Stress), and multiplying them by 2, but it is also possible to compute a composite score of ‘General Distress’ resulting from the sum of all items. Accordingly, total scale scores may range between 0 and 63 and subscales may range between 0 and 42.
- Perceived Stress Scale (PSS) [62,63]: The PSS is a self-report questionnaire for evaluating individuals’ perceived stress. The scale is composed of 10 items on a 5-point Likert, and it measures to what extent our daily experiences are subjectively perceived as stressful in the last month (e.g., ‘How often you have been upset because of something that happened unexpectedly?’) For the current study, it was adapted to evaluate the perceived level of stress in the last week. It yields a composite score of perceived stress resulting from the sum of the responses of single items, from 0 to 40.
- Beck Hopelessness Scale (BHS) [64,65]: The BHS is a self-report questionnaire for measuring pessimistic thoughts or negative attitudes toward the future in three different life spheres: feelings about the future, loss of motivation, and general expectations. It is composed of 20 true–false items. The total sum score can range from 1 to 20, with higher scores underscoring higher levels of hopelessness.

#### 2.3.2. Secondary Outcome Measures

Participants were also assessed at four time points with the following measures:

- Social Connectedness Scale (SCS) [66,67]: The SCS is a self-report instrument that measures the extent to which the individual feels connected to other persons or the social context. The scale is composed of 8 items on a 6-point Likert scale. Sum scores can range from 0 to 48, with higher scores indicating a higher sense of social connectedness.
- Fear of Coronavirus (FCOR) [68,69]: FCOR is a scale aimed at measuring the level of fear experienced during the COVID-19 pandemic (‘I am most afraid of coronavirus-19’). FCOR is composed of 8 items on a 5-point scale. Sum scores can range from 0 to 40, with higher scores indicating higher fear of COVID-19.

#### 2.3.3. State Outcome Measures

Furthermore, the following self-report questionnaires were administered each day to measure changes in daily subjective experience from the waiting period (7 days before the start of the intervention) and after the completion of each daily session (from Day 0 to Day 7):

- State–Trait Anxiety Inventory (STAI) [70,71]: The STAI is a 40-item self-report questionnaire on a 4-point Likert scale where participants are asked to evaluate both state and trait levels of anxiety. This questionnaire is divided into 20 items that refer to state anxiety (STAI-S), and 20 items that refer to trait anxiety (STAI-T). In this study, we used only the STAI-S. Total scores range from 20 to 80, where higher scores indicate higher anxiety feelings.
- Smith Relaxation State Inventory 3 (SRSI3) [72]: The SRSI3 is the revised version of the Smith Relaxation State inventory and measures 19 relaxation states. Individuals are asked to evaluate how they feel right now in response to 38 items on a 6-point Likert scale. The scale is divided into four subscales that can be selected independently. For this protocol, the following subscales have been selected, for a total of 20 items: rest/refresh, energised, physical relaxation, at ease/peace, joy, mental quiet, awareness, somatic stress, emotional stress, and cognitive stress.
- Subjective Units of Distress Scale (SUDS) [73]: The SUDS is a numeric rating scale from 0 to 100 that evaluates the subjectively perceived level of distress.

### 2.4. Data Analysis

Sample size calculation was determined using G*Power (3.1) with a medium effect size (f = 0.25) [74], a power of 0.95, and an alpha of 0.05. We expected a medium effect size considering a recent meta-analysis of digital interventions for stress management in adults underlying that for the primary outcome of stress, an effect size of Cohen d = 0.43 was found across the 26 comparisons [75]. For a mixed between- and within-subject design (2 × 4), a minimum total sample size of 36 was suggested. Linear mixed-effects models (LMMs, estimated using REML and nloptwrap optimiser) were fitted to evaluate the effectiveness of the intervention. These models estimate both interindividual variability and intraindividual patterns of change over repeated sessions, thus controlling the issue of non-independence among repeated measures. Two separate models were fitted to evaluate changes for each outcome score (DASS-21_Depression, DASS-21_Anxiety, DASS-21_Stress, DASS-21_General Distress, PSS, BHS, SCS, FCOR) across four time points from the waiting period (7 days before the start of the treatment—Waiting Period) to the follow-up phase (T2). The first model was run according to the following formula = outcome ~ time, and the second one according to this formula = outcome ~ time*Immersion, to check for the potential effect on the results of using an immersive vs. a non-immersive modality. As reported in Table 1, 18 participants accessed the protocol through their smartphone and a basic low-cost cardboard VR headset (‘immersive modality’), and 22 participants followed the intervention through the YouTube App (‘non-immersive modality’). All models included participants as a random effect (formula = ~1 | ID). Table S1 (Supplementary Materials) presents the parameters estimated for each model that was fitted. In model comparisons, we selected the most parsimonious model according to Akaike’s information criteria (AIC) and Bayesian information criteria (BIC) to evaluate which model resulted to better fit the data. Then, we applied the same pipeline for evaluating changes in the daily subjective experience (STAI-S, SRSI3-Rest/Refresh, SRSI3-Energised, SRSI3-Physical relaxation, SRSI3-At ease/peace, SRSI3-Joy, SRSI3-Mental quiet, SRSI3-Aware, SRSI3-Somatic stress, SRSI3-Emotional stress, SRSI3-Cognitive stress, and SUDS) between the waiting period and the treatment week. Daily measures were first averaged to obtain a measure of global subjective experience before the starting of the treatment (waiting-list period) and after the treatment sessions (treatment period). Table S2 (Supplementary Materials) shows the parameters estimated for each model that was fitted. Analyses of variance (ANOVA) on selected LMM parameters were performed with Kenward–Roger approximation for degrees of freedom [76]. Significant effects were examined with post hoc comparisons (Bonferroni’s adjustment) and were performed with emmeans and are reported with estimated marginal means and standard error (SE). Assumptions for all models were satisfied. The linear mixed models were run in R Studio Version 1.1.463 with the following packages: Lme4 [77], with restricted maximum likelihood [78], lmerTest [79], emmeans [80]. Then, clinical change analyses on the primary outcome measures were carried out using the reliable change index (RCI) and the clinically significant change (CSC) (with formulas explained here [81]). RCI is an objective measure of change magnitude that accounts for errors of measurement, namely, the index explains if the participant improved (or worsened) between her/his pre–post-test questionnaire scores and if this improvement is not due to the measurement error. The CSC explains if the reliable change could be considered as important from a clinical point of view for the individual providing a cutoff for the clinical change (i.e., the score must pass the cutoff from pre- to post-test). Available Italian normative data for the DASS-21 [61] and PSS [63] were used to compute the indices. PSS scores are calculated for males and females separately, as normative data do not provide global measures of the population. The normative data of each questionnaire were used to compute the RCI and the CSC for each individual between pre- and post-test scores. Consequently, RCI and CSC indexes for each participant were calculated from the DASS-21 subscales and the PSS (males and females) normative data. Pre- and post-measures were taken from the waiting week (i.e., no intervention) and the seven days of treatment regardless of the level of immersion. 

By counting the number of individuals with a positive or negative reliable change (i.e., RCI), risk measures and effect size of the intervention were computed [82,83]. Relative risk (RR) is a proportional measure estimating the size of the risk in the intervention period. Values close to 0 indicate a protective effect of the intervention (i.e., the risk of a bad outcome is reduced by the treatment). Experimental and control event ratios (EER, CER) indicate the incidence of a negative outcome in the two phases (intervention week vs. waiting period) in percentage (i.e., the lower the better in this case). The relative risk reduction (RRR) indicates the extent the intervention reduced the risk of bad outcomes relative to the waiting period in percentage (i.e., the higher the better in this case). Absolute risk reduction (ARR) indicates the percentage of participants who would be prevented from developing bad outcomes (i.e., the higher the better in this case). The number needed to treat (NNT) indicates the number of participants to treat to prevent a negative outcome (i.e., here the lower the better).

## 3. Results

### 3.1. Primary and Secondary Outcome Measures

Table 2 displays the means for primary and secondary outcomes across each assessment. None of the models were significant on the factor ‘immersion’, suggesting that there is no significant effect of the modality (immersive vs. non-immersive) on the results of the treatment. Furthermore, the models that include the factor immersion reported higher values of AIC and BIC parameters, suggesting that the models according to this formula: =outcome ~ time, were the best fit for the data (see Table S1).

**Figure 3:**
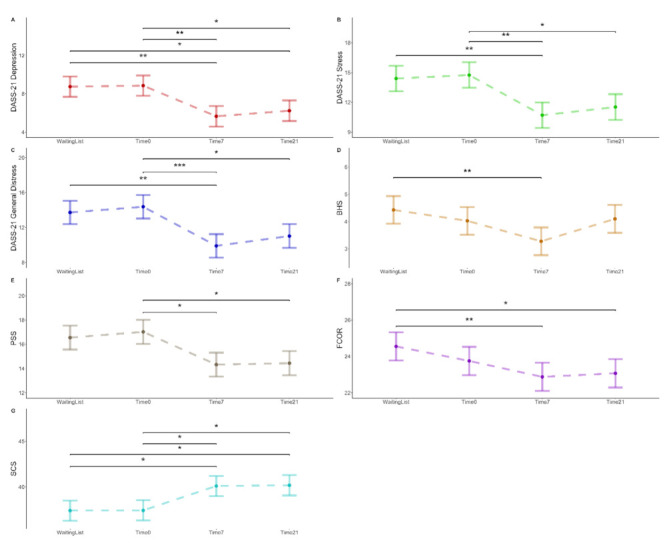
Bonferroni-adjusted, pairwise comparisons for Depression Anxiety Stress Scale Subscale Depression (DASS_21 Depression, (**A**) and Subscale Stress (DASS_21 Stress, (**B**), Depression Anxiety Stress Scale total score General Distress (DASS_21 General Distress, (**C**), Beck Hopelessness Scale (BHS, (**D**)), Stress, Perceived Stress Scale (PSS, (**E**)), Fear of Coronavirus outcome (FCOR, (**F**)), and Social Connectedness Scale (SCS, (**G**)) across the different time points. (7 days before the start of the intervention—Waiting Period, before the start of the intervention, Day 0—T0; end of the intervention, Day 7—T1; 2-week follow-up, Day 21—T2). * <0.05; ** <0.01; *** <0.001.

Analysis of variance (ANOVA) on the LMM’s parameters indicated a significant effect of time for the primary outcomes of depression [F(3, 115.2) = 6.68; *p* < 0.001, η^2^_p_ = 0.15, 95% CI (0.04, 0.26)], stress [F(3, 115.17) = 6.35; *p* < 0.001, η^2^_p_ = 0.14, 95% CI (0.03, 0.25)], general distress [F(3, 115.15) = 6.97; *p* < 0.001, η^2^_p_ = 0.15, 95% CI (0.04, 0.26)], perceived stress F(3, 115.17) = 5.15; *p* = 0.002, η^2^_p_ = 0.12, 95% CI (0.02, 0.22)], and hopelessness [F(3, 115.07) = 4.80, *p* = 0.003, η^2^_p_ = 0.11, 95% CI (0.01, 0.21)]. No significant effect of time was found on anxiety, as measured by DASS-21 [F(3, 115.2) = 2.2556, *p* > 0.05, η^2^_p_ = 0.06, 95% CI (0.00, 0.14)]. Among the secondary outcomes, ANOVA on the linear mixed model’s parameters indicated a significant effect of time on social connectedness [F(3, 115.15) = 5.48, *p* = 0.001, η^2^_p_ = 0.12, 95% CI (0.02, 0.23)] and fear of coronavirus [F(3, 115.07) = 5.0214, *p* = 0.003, η^2^_p_ = 0.12, 95% CI (0.02, 0.22)]. All these significant effects were followed up with post hoc (Bonferroni adjusted) comparisons testing to determine changes in outcomes across the different time points. See Figure 3 for a summary of these findings. Full comparisons are presented in Supplementary Materials (see Table S3). 

First, results indicated no significant changes (*p* = 1.000) for all the measures collected between the waiting period (i.e., data collected 7 days before the start of the treatment) and the baseline (i.e., data collected at the beginning of the intervention, T0). Regarding primary outcome measures, participants exhibited improvements from baseline to post-intervention for depression levels, stress levels, general distress, and perceived stress (all *p*s < 0.05) but not for the perceived hopelessness (*p* = 0.110). Results for the secondary outcomes indicated an increase in social connectedness from T0 to T1 (*p* = 0.033) but not a significant reduction in perceived fear of coronavirus (*p* = 0.412). Among these study variables, significant improvements observed from T0 and T1 were maintained from post-intervention to the 2-week follow-up (*p* > 0.05). Levels of depression, stress, general distress, and perceived stress significantly decreased from T0 to T2, and an increase in perceived interpersonal closeness with the social world was also observed (all *p*s < 0.05).

### 3.2. State Outcome Measures

**Figure 4:**
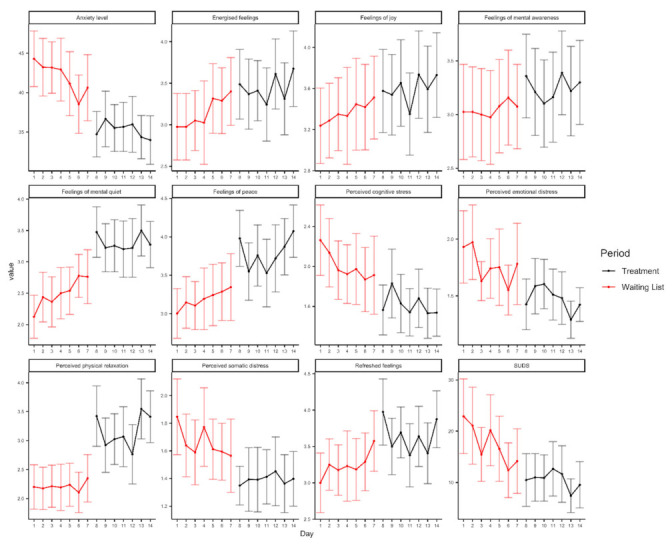
Daily changes in the subjective experience (refreshed feelings, SRSI3-Rest/Refresh; energised feelings; SRSI3-Energised, relaxation SRSI3-Physical relaxation; feelings of peace, SRSI3-At ease/peace; feelings of joy, SRSI3-Joy; mental quiet, SRSI3-Mental quiet; mental awareness, SRSI3-Aware; somatic stress, SRSI3-Somatic stress; emotional stress, SRSI3- Emotional stress; cognitive stress, SRSI3-Cognitive stress; anxiety level, State–Trait Anxiety Inventory-State—STAI-S; discomfort, Subjective Units of Distress Scale SUDS). State measures collected each day during the waiting week (7 days before the start of the intervention) and during the intervention phase, after each treatment module. Mean and 95% CI are depicted in the graphs.

Table 3 displays the means for the state outcome measures collected each day during the waiting week (7 days before the start of the intervention) and during the intervention phase, after each treatment module. Daily changes in the subjective experience across the two time periods are presented in Figure 4. Globally, the percentage of missing responses did not exceed 5% (We handled missing data averaging scores across weeks. Moreover, we also tried to impute values with a random forest algorithm (mice package in R), but the trends of the measures did not substantially change (see also Figure S1)). An improvement in almost all study variables was observed from the waiting period (i.e., data collected each day, for 7 days before the start of the intervention) to the intervention phase, namely, data collected each day after each treatment module. None of the models were significant on the factor ‘immersion’, suggesting that there is no significant effect of type of modality (immersive vs. non immersive) on the results of the treatment. Furthermore, the models that include the factor immersion reported higher values of AIC and BIC parameters, suggesting that the models according to this formula: =outcome ~ time, were the best fit for the data. Regarding the model including the subscale SRSI3- Aware, results indicated no effect from time or immersion (see Table S2). Analysis of variance (ANOVA) on the LMM’s parameters indicated a significant effect of time on anxiety levels [F(1, 39.323) = 39.99; *p* < 0.001, η^2^_p_ = 0.50, 95% CI (0.28, 0.66)], refreshed feelings [F(1, 39.732) = 6.35; *p* = 0.007, η^2^_p_ = 0.17, 95% CI (0.01, 0.38)], energised feelings [F(1, 39.836) = 5.54; *p* = 0.023, η^2^_p_ = 0.12, 95% CI (0.00, 0.32)], perceived physical relaxation F(1, 39.634) = 25.75; *p* < 0.001, η^2^_p_ = 0.39, 95% CI (0.16, 0.57)], feelings of peace [F(1, 40.242) = 5.23, *p* = 0.027, η^2^_p_ = 0.12, 95% CI (0.00, 0.32)], feelings of mental quiet [F(1, 39.558) = 31.36, *p* < 0.001, η^2^_p_ = 0.44, 95% CI (0.21, 0.61)], perceived somatic distress [F(1, 39.156) = 31.59, *p* < 0.001, η^2^_p_ = 0.45, 95% CI (0.21, 0.61)], perceived emotional distress [F(1, 39.348) = 15.36, *p* < 0.001, η^2^_p_ = 0.28, 95% CI (0.07, 0.48)], perceived cognitive distress [F(1, 39.178) = 31.51, *p* < 0.001, η^2^_p_ = 0.45, 95% CI (0.21, 0.61)], and subjectively perceived levels of discomfort [F(1, 39.206) = 25.59, *p* < 0.001, η^2^_p_ = 0.39, 95% CI (0.16, 0.57)]. No significant effect of time emerged for perceived feelings of joy [F(1, 39.285) = 3.83, *p* = 0.057, η^2^_p_ = 0.09, 95% CI (0.00, 0.29)].

### 3.3. Clinical Change Analyses of Primary Outcomes

Table 4 provides the results obtained from the clinical change analyses of primary outcome measure scales. From a clinical point of view, all the scales indicated good outcomes and low risk for the intervention period, compared to the waiting one. The total number of pre/post-tests with a positive RCI was 29 for the treatment period, compared to 10 in the waiting period. These results indicate that almost 73% of the participants in the intervention week improved on the post-intervention measures. To understand if this change could be considered clinically significant, the analysis revealed that 16 out of the 29 RCI pre/post-tests reached the CSC, whereas only 3 out of 10 reached the cutoff for clinical change in the waiting period. These results indicate that more than 50% of the participants in the intervention period reported an improvement that could be considered clinically significant. Conversely, the pre/post-tests with a negative RCI were 4 in the treatment period, compared to 20 in the waiting one, highlighting a protective effect of the treatment.

**Figure 5:**
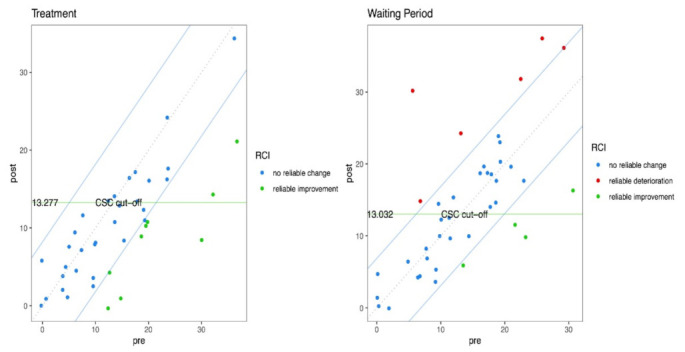
Jacobson plot depicting the 95% CI of the RCI between the blue lines and the CSC cutoff for pre- and post-test between the green lines. Blue dots represent participants who did not have a reliable change between pre and post measures. Red dots represent individuals who had a reliable deterioration between the two time points. Green dots are participants who showed a reliable improvement between the two measures. Green dots falling in the bottom-right section of the rectangle delimited by the green horizontal and vertical lines are participants with a reliable change plus a CSC. Green dots falling in the bottom-left section of the rectangle delimited by the green horizontal, and vertical lines are participants who already have high scores to show a CSC. RCI: reliable change index; CSC: clinically significant change.

The number of adverse outcomes during the first seven days of treatment compared to the waiting list (i.e., 7 days before the start of the intervention) is lower in all the relevant scales, including also the DASS-21-Anxiety that was found to be not statistically significant. These results are supported by the different scores of risks included in Table 4. Regarding the scales analysed, the risk associated with the treatment ranged between 0 to 0.44. The incidence of negative outcomes in the treatment period (EER) was between 0% and 33%, whereas the incidence of negative outcomes in the waiting period (CER) was from 40% to 100%. The reduction of risk in the experimental period (RRR) ranged from 56% to 100% and 29% to 100% of the participants treated would be prevented from developing negative outcomes (ARR). Taking into consideration the DASS-21, the best outcomes were observed for the total score, the general distress (see also Figure 5). Despite a small effect size, the depression subscale showed good protective effects of the treatment compared to the waiting period. Interestingly the anxiety subscale (DASS-21-Anxiety), despite not being statistically significant, highlighted a medium effect size of the treatment with good protective effects, compared to the waiting period. For the PSS, the males showed lower risk associated with the intervention compared to females; however, the effect size was lower for the former group, compared to the latter, and the two samples are small and unbalanced. 

## 4. Discussion

The aim of this study was to evaluate the effectiveness of a novel virtual reality (VR) self-help psychological protocol to assist individuals in coping with the psychological burden related to the COVID-19 pandemic and associated social restriction measures. The entire protocol (1 week) is based on the 360° video (VR) ‘The Secret Garden’, which simulates a natural environment aiming to promote relaxation and self-reflection. ‘The Secret Garden’ experience is combined with daily social exercises with targeted goals that are designed to be experienced with another person. The sample consisted of 40 Italian participants who experienced at least two months of strict social distancing measures, including a ‘stay-at-home’ order, implemented by the Italian National Government to contain the dramatic diffusion of the disease in the country (the so-called Fase-Uno). To evaluate the effectiveness of the protocol we compared changes in levels of depression, anxiety, stress, general distress, perceived stress, hopelessness, social connectedness, and fear of COVID-19 between a control waiting period with changes before the start of the treatment, at the end of the treatment period, and at 2-week follow-up.

First, results indicated a significant change between the different time points (waiting period vs. baseline assessment vs. end of intervention vs. 2-week of follow-up) on all outcome measures, except for the anxiety subscale of the Depression Anxiety Stress Scale (DASS_21). Importantly, results indicated no significant changes for these measures between the waiting period (i.e., data collected 7 days before the start of the treatment) and the baseline (i.e., data collected at the beginning of the intervention), but from the baseline assessment and the end of the intervention.

Regarding primary outcome measures, participants exhibited improvements in depression levels, stress levels, general distress, and perceived stress but not for the perceived hopelessness. Results for the secondary outcomes indicated an increase in social connectedness but not a significant reduction in perceived fear of COVID-19. Results revealed that these treatment gains were maintained in the 2-weeks of follow-up.

Regarding the lack of changes in perceived anxiety levels (as measured with the DASS_21), although we observed a decrease from the baseline to post-intervention, we also measured a slight increase in scores from the waiting period to the start of the protocol. This slight increase in the perceived anxiety from the control period to the start of the treatment could be explained by data reviewed by recent meta-analyses indicating a high prevalence (23.4%) of anxiety symptoms among the general population measured with the DASS-21 scale during the COVID-19 pandemic [84]. Additionally, we did not observe any effect of the intervention on the subjective perception of hopelessness. An explanation might be offered by Cipolletta and Ortu [85], who suggested that one of the most profound psychological effects of the COVID-19 crisis and related restrictive measures is that this period has suspended time and our future [85], with a significant rebound in feelings of hopelessness and demoralisation [19]. 

Regarding the secondary outcome measures, we found a significant improvement in the feeling of social connectedness with the social world, but we did not find a reduction in the fear of COVID-19. Globally, we observed a reduction of fear of COVID-19 from the waiting period to the follow-up assessment, but the observed decrease from the beginning and the end of the intervention was not statistically significant. This finding could be explained by taking into consideration the nature of the COVID Feel Good intervention, i.e., it was specifically developed to offer participants a self-help tool for overcoming the psychological distress associated with the COVID-19 pandemic but without a specific reference to the pandemic itself. Accordingly, it is possible to observe that threat of catching the virus remained stable across the different assessment periods (see Table 2). We have indeed exploited the potentiality of VR as a ‘simulative instrument’ to provide participants with the opportunity to be immersed in a naturalistic and safe digital place [56], far from the stressful situations experienced in routine daily contexts during the pandemic, where they can learn how to relax and reflect upon their experience following a guided protocol. This effect has been enhanced by creating a bridge with the real-life context with different daily social tasks aimed at facilitating a process of critical examination and eventually revision of core assumptions and beliefs [54]. 

Wahlund et al. [86] offered another example of a brief self-guided, online psychological intervention. In this case, the intervention was specifically developed for targeting dysfunctional COVID-19 worry and associated symptoms. Consequently, they found a reduction in COVID-19-related worry for the intervention group compared to the waiting list, and improvements on all secondary outcome measures, including mood, daily functioning, insomnia, and intolerance of uncertainty.

As regards daily changes, we observed an improvement in the daily levels of anxiety, perceived stress, and mood levels from the waiting period to the treatment week, further suggesting the effectiveness of each module in ameliorating participants’ subjective experience in their daily context. 

Importantly, these improvements are observed both at the statistical and clinical level. Indeed, we also explored the magnitude of the clinical change associated with the COVID Feel Good protocol with a series of clinical change analyses on the primary outcome measures of DASS_21 subscales and PSS. Our results indicated that the intervention was associated with good clinical outcomes, low-to-no risks for the intervention, and no adverse effects. Specifically, regarding the treatment period, we observed a positive change (defined as an improvement between the pre- and post-test scores in the self-reported questionnaires) in 73% of cases and among these, 50% of the participants reported a significant clinical improvement. 

In sum, evidence suggests a potential benefit of the intervention in reducing psychological distress during a life-threatening situation. In this perspective, it is crucial to reiterate that participants had the opportunity to follow the protocol with the use of a head-mounted display (HMD, immersive modality) or without it, namely, by watching the 360° video entitled ‘The Secret Garden’ on Youtube (non-immersive modality). We did not find any difference in all outcome measures as regards the immersion, suggesting the potential applicability of the intervention in populations with no direct access to advanced technological devices, such as the use of an HMD. 

These initial findings on the effectiveness of COVID Feel Good training contribute to the growing literature supporting the emerging role of digital self-help interventions in addressing mental health symptoms and promoting well-being [42,43,44,45,46,47]. As the countries and societies will hopefully begin to emerge from social distancing measures, a further step will include investigating whether this novel self-help intervention would ameliorate participants’ mental well-being without the peculiar conditions of a lockdown. Several studies have emphasised that a pandemic could have long-term adverse psychological sequelae such as anxiety, depression, post-traumatic stress among survivors, health professionals, but also the general population [87]. Continuous research efforts are needed to both develop and test effective evidence-based self-help interventions for improving mental health in the near future [88].

### Limitations

Given the importance of delivering and testing digital psychological treatments to help individuals cope with psychological distress related to the COVID-19 crisis [38,89], the results obtained might have important implications for developing and testing future evidence-based protocols in this field. However, it is important to note that this study also has several limitations. First, as noted earlier, although our study was not aimed at comparing the use of immersive vs. non-immersive in VR-based self-help treatments, we did not experimentally control for this variable. Future research could aim to specifically examine the impact of the level of immersion on the delivery of digital psychological self-help treatments. Second, given the specific context of the intervention, we opted to not include an active control group, but rather offered all participants the possibility to participate in the study. The absence of a proper control condition may have impacted the results. Future studies should be carried out to further investigate the efficacy of COVID Feel Good in comparison to standard psychological treatments. Third, the participation in the protocol was limited to individuals with proper access to a smartphone with internet access and availability. This might have impacted the generalisability of the results obtained. Finally, the reported findings only measured short-term outcomes, and it is crucial to acknowledge that a 2-week follow-up is not adequate to properly draw long-term conclusions. Future larger controlled and randomised trials including a long-term follow-up should evaluate the clinical efficacy of this novel self-help intervention in ameliorating the psychological distress associated with the COVID-19 crisis. 

## 5. Conclusions

The psychological and social costs of the COVID-19 pandemic are now evident. If not treated promptly and properly with appropriate evidence-based interventions, the psychological burden of COVID-19 may have long-term negative health effects. Globally, this study has important implications for the design and delivery of digital self-help psychological protocols to alleviate the psychological distress associated with the pandemic [7,8]. This effectiveness study tried to investigate if and how a daily VR self-help protocol can help overcome the psychological burden associated with the COVID-19 crisis and related social restrictive measures. The results, even if preliminary, suggest a potential benefit of the intervention in reducing psychological distress in participants who had experienced at least two months of strict social distancing measures. Moreover, its current availability [90] in 12 different languages—English, Spanish, French, Brazilian/Portuguese, German, Italian, Turkish, Japanese, Korean, Farsi, Romanian, and Catalan—makes COVID Feel Good a free choice for assisting individuals worldwide to cope with the psychological distress related to the COVID-19 pandemic and related restrictions. However, randomised controlled trials are needed to evaluate the clinical efficacy of this protocol in large samples, in other groups (i.e., health professionals or older adults), and its impact in the longer term. 

## Figures and Tables

**Table 1:** Demographic characteristics of participants.

Variables	Total Sample (N = 40)
Age (years), mean (SD)	30.28 (11.69)
Gender, N (%)	
Male	15 (37.5)
Female	25 (62.5)
Relationship Status, N (%)	
Single	30 (72.5)
Married	10 (25)
Employment Status, N (%)	
Student	22 (55)
Employed	18 (45)
Housing arrangements, N (%)	
Living alone	<5 (<5)
Living with partner	6 (15)
Living with partner and children	5 (12.5)
Living with parents	23 (57.5)
Living with friends	<5 (<5)
Other	<5 (<5)
Experience of COVID Feel Good, N (%)	
Immersive modality Non-Immersive modality	18 (45) 22 (55)

**Table 2:** Descriptive statistics for the primary and outcome measure across the different time points by time points (7 days before the start of the intervention—Waiting Period, before the start of the intervention, Day 0—T0; end of the intervention, Day 7—T1; 2-week follow-up, Day 21—T2). Data are provided in means and standard deviation (SD). Depression Anxiety Stress Scale Subscale Depression-21: DASS_21; Perceived Stress Scale: PSS; Beck Hopelessness Scale: BHS; Social Connectedness Scale: SCS; Fear of Coronavirus outcome: FCOR.

		Waiting Period Mean (SD)	T0 Mean (SD)	T1 Mean (SD)	T2 Mean (SD)
Primary outcome measures					
DASS-21	Depression symptoms	8.75 (6.80)	8.85 (8.18)	5.65 (5.51)	6.37 (6.59)
	Anxiety symptoms	4.30 (3.88)	5.15 (4.77)	3.45 (4.51)	4.21 (4.50)
	Stress symptoms	14.40 (8.25)	14.75 (8.14)	10.70 (7.01)	11.74 (9.10)
	General distress	13.73 (7.85)	14.44 (9.34	9.90 (7.08)	11.23 (9.56)
PSS	Perceived stress level	16.55 (5.82)	17.03 (6.18)	14.33 (5.98)	14.50 (6.97)
BHS	Perceived hopelessness	4.42 (3.22)	4.03 (3.08)	3.27 (2.79)	4.13 (3.71)
Secondary outcome measures					
SCS	Perceived social connectedness	37.40 (7.81)	37.43 (6.63)	40.10 (6.64)	40.08 (6.96)
FCOR	Fear of coronavirus	24.55 (4.62)	23.75 (4.74)	22.88 (5.00)	23.03 (5.36)

**Table 3:** Descriptive statistics for the state measures collected each day during the waiting week (7 days before the start of the intervention) and during the intervention phase, after each treatment module. Data are provided in means and standard deviation (SD). State–Trait Anxiety Inventory-State: STAI-S; Smith Relaxation State Inventory 3: SRSI3; Subjective Units of Distress Scale: SUDS.

	State Outcome Measures	Waiting Period	Intervention Week
		Mean (SD)	Mean (SD)
STAI-S	Anxiety level	42.00 (9.48)	35.29 (7.52)
SRSI3	Refreshed feelings	3.24 (0.69)	3.67 (0.88)
	Energised feelings	3.09 (0.60)	3.46 (1.00)
	Perceived physical relaxation	2.22 (0.87)	3.15 (1.25)
	Feelings of peace	3.20 (0.79)	4.76 (0.41)
	Feelings of joy	3.38 (0.91)	3.61 (1.02)
	Feelings of mental quiet	2.50 (0.81)	3.31 (0.96)
	Feelings of mental awareness	3.06 (1.01)	3.25 (0.96)
	Perceived somatic distress	1.66 (0.55)	1.40 (0.50)
	Perceived emotional distress	1.76 (0.66)	1.48 (0.46)
	Perceived cognitive stress	2.01 (0.83)	1.62 (0.67)
SUDS	Subjectively perceived levels of discomfort	17.62 (15.6)	10.71 (13.1)

**Table 4:** Clinical change analyses for the primary outcome measures. RCI indicates the number of participants who improved (improvement RCI) or worsened (deterioration RCI) from the waiting period to the treatment period for the relevant scales. CSC shows the number of participants that reached a clinical change in the selected scales. RCI is used to calculate risk and efficacy measures. RCI: reliable change index; CSC: clinically significant change; RR: relative risk; CER: control event rate; EER: experimental event rate; RRR: relative risk reduction; AAR: absolute risk reduction; NNT: number needed to treat. Measures from CER to NNT are expressed in percentage.

Variables	Improvement RCI	Deterioration RCI	Improvement CSC	RR	CER	EER	RRR	ARR	NNT	Effect Size *d*
Depression Symptoms	Treatment: 4 Waiting period: 2	Treatment: 0 Waiting period: 4	Treatment: 1 Waiting period: 0	0	67	0	100	67	1	0.45 small-beneficial
Anxiety Symptoms	Treatment: 4 Waiting period: 0	Treatment: 2 Waiting period: 4	Treatment: 3 Waiting period: 0	0.33	100	33	67	67	1	0.56 medium-beneficial
Stress symptoms	Treatment: 8 Waiting period: 3	Treatment: 1 Waiting period: 2	Treatment: 4 Waiting period: 0	0.28	40	11	72	29	3	0.55 medium-beneficial
General Distress	Treatment: 9 Waiting period: 4	Treatment: 0 Waiting period: 6	Treatment: 5 Waiting period: 2	0	60	0	100	60	2	0.6 medium-beneficial
Perceived Stress Level	Treatment: 2 Waiting period: 1	Treatment: 1 Waiting period: 3	Treatment: 2 Waiting period: 1	0.44	75	33	56	42	2	0.66 medium-beneficial
Perceived Stress Level	Treatment: 2 Waiting period: 0	Treatment: 0 Waiting period: 1	Treatment: 1 Waiting period: 0	0	100	0	100	100	1	0.22 small-beneficial

## Data Availability

The data presented in this study are available on request from the corresponding author.

## Supplementary Materials

The following are available online at https://www.mdpi.com/article/10.3390/ijerph18158188/s1, Figure S1: Results of imputation with random forest algorithm compared to real values; Table S1: Estimated parameters for Linear Mixed Models fitted for primary outcome measures; Table S2: Estimated parameters for Linear Mixed Models fitted for state outcome measures; Table S3: Bonferroni adjusted-pairwise comparisons for all primary and secondary outcome measure across the different time points.

## References

[1] Paules C.I., Marston H.D., Fauci A.S. (2020). Coronavirus Infections-More Than Just the Common Cold. JAMA J. Am. Med. Assoc..

[2] Reiss C.S. (2020). Coronavirus Pandemic. DNA Cell Biol..

[3] Jee Y. (2020). WHO International Health Regulations Emergency Committee for the COVID-19 outbreak. Epidemiol. Health.

[4] Remuzzi A., Remuzzi G. (2020). COVID-19 and Italy: What next?. Lancet.

[5] Katz R., Vaught A., Simmens S.J. (2019). Local Decision Making for Implementing Social Distancing in Response to Outbreaks. Public Health Rep..

[6] Brooks S.K., Webster R.K., Smith L.E., Woodland L., Wessely S., Greenberg N., Rubin G.J. (2020). The psychological impact of quarantine and how to reduce it: Rapid review of the evidence. Lancet.

[7] Holmes E.A., O’Connor R.C., Perry V.H., Tracey I., Wessely S., Arseneault L., Ballard C., Christensen H., Cohen Silver R., Everall I. (2020). Multidisciplinary research priorities for the COVID-19 pandemic: A call for action for mental health science. Lancet Psychiatry.

[8] Vindegaard N., Benros M.E. (2020). COVID-19 pandemic and mental health consequences: Systematic review of the current evidence. Brain. Behav. Immun..

[9] Marazziti D., Pozza A., Di Giuseppe M., Conversano C. (2020). The psychosocial impact of COVID-19 pandemic in Italy: A lesson for mental health prevention in the first severely hit European country. Psychol. Trauma Theory Res. Pract. Policy.

[10] Pfefferbaum B., North C.S. (2020). Mental health and the Covid-19 pandemic. N. Engl. J. Med..

[11] Pagnini F., Bonanomi A., Tagliabue S., Balconi M., Bertolotti M., Confalonieri E., Di Dio C., Gilli G., Graffigna G., Regalia C. (2020). Knowledge, Concerns, and Behaviors of Individuals during the First Week of the Coronavirus Disease 2019 Pandemic in Italy. JAMA Netw. Open.

[12] De Giorgio A. (2020). Global Psychological Implications of Severe Acute Respiratory Syndrome Coronavirus 2 (SARS-CoV-2) and Coronavirus Disease-2019 (COVID-19). What Can Be Learned From Italy. Reflections, Perspectives, Opportunities. Front. Psychol..

[13] Xiong J., Lipsitz O., Nasri F., Lui L.M.W., Gill H., Phan L., Chen-Li D., Iacobucci M., Ho R., Majeed A. (2020). Impact of COVID-19 pandemic on mental health in the general population: A systematic review. J. Affect. Disord..

[14] Franceschini C., Musetti A., Zenesini C., Palagini L., Scarpelli S., Quattropani M.C., Lenzo V., Freda M.F., Lemmo D., Vegni E. (2020). Poor sleep quality and its consequences on mental health during the COVID-19 lockdown in Italy. Front. Psychol..

[15] Rubin G.J., Wessely S. (2020). The psychological effects of quarantining a city. BMJ.

[16] Rossi R., Socci V., Talevi D., Mensi S., Niolu C., Pacitti F., Di Marco A., Rossi A., Siracusano A., Di Lorenzo G. (2020). COVID-19 Pandemic and Lockdown Measures Impact on Mental Health Among the General Population in Italy. Front. Psychiatry.

[17] Park S.C., Park Y.C. (2020). Secondary emotional reactions to the Covid-19 outbreak should be identified and treated in Korea. J. Korean Med. Sci..

[18] Fernández R.S., Crivelli L., Guimet N.M., Allegri R.F., Pedreira M.E. (2020). Psychological distress associated with COVID-19 quarantine: Latent profile analysis, outcome prediction and mediation analysis. J. Affect. Disord..

[19] Gruber J., Prinstein M.J., Clark L.A., Rottenberg J., Abramowitz J.S., Albano A.M., Aldao A., Borelli J.L., Chung T., Davila J. (2020). Mental Health and Clinical Psychological Science in the Time of COVID-19: Challenges, Opportunities, and a Call to Action. Am. Psychol..

[20] Ruffolo M., Price D., Schoultz M., Leung J., Bonsaksen T., Thygesen H., Geirdal A.Ø. (2021). Employment Uncertainty and Mental Health During the COVID-19 Pandemic Initial Social Distancing Implementation: A Cross-national Study. Glob. Soc. Welf..

[21] Rettie H., Daniels J. (2020). Coping and Tolerance of Uncertainty: Predictors and Mediators of Mental Health During the COVID-19 Pandemic. Am. Psychol..

[22] Satici B., Saricali M., Satici S.A., Griffiths M.D. (2020). Intolerance of Uncertainty and Mental Wellbeing: Serial Mediation by Rumination and Fear of COVID-19. Int. J. Ment. Health Addict..

[23] Thorell L.B., Skoglund C., de la Peña A.G., Baeyens D., Fuermaier A.B.M., Groom M.J., Mammarella I.C., van der Oord S., van den Hoofdakker B.J., Luman M. (2021). Parental experiences of homeschooling during the COVID-19 pandemic: Differences between seven European countries and between children with and without mental health conditions. Eur. Child Adolesc. Psychiatry.

[24] Sinclair R.R., Probst T.M., Watson G.P., Bazzoli A. (2021). Caught between Scylla and Charybdis: How economic stressors and occupational risk factors influence workers’ occupational health reactions to COVID-19. Appl. Psychol..

[25] Townsend A.K., Hawley D.M., Stephenson J.F., Williams K.E.G. (2020). Emerging infectious disease and the challenges of social distancing in human and non-human animals. Proc. R. Soc. B Biol. Sci..

[26] Riva G., Wiederhold B.K., Mantovani F. (2021). Surviving COVID-19: The Neuroscience of Smart Working and Distance Learning. Cyberpsychol. Behav. Soc. Netw..

[27] Sutin A.R., Luchetti M., Terracciano A. (2020). Has loneliness increased during COVID-19? Comment on “Loneliness: A signature mental health concern in the era of COVID-19”. Psychiatry Res..

[28] Rodriguez L.M., Litt D.M., Stewart S.H. (2020). Drinking to cope with the pandemic: The unique associations of COVID-19-related perceived threat and psychological distress to drinking behaviors in American men and women. Addict. Behav..

[29] Magson N.R., Freeman J.Y.A., Rapee R.M., Richardson C.E., Oar E.L., Fardouly J. (2021). Risk and Protective Factors for Prospective Changes in Adolescent Mental Health during the COVID-19 Pandemic. J. Youth Adolesc..

[30] Ye Z., Yang X., Zeng C., Wang Y., Shen Z., Li X., Lin D. (2020). Resilience, Social Support, and Coping as Mediators between COVID-19-related Stressful Experiences and Acute Stress Disorder among College Students in China. Appl. Psychol. Heal. Well-Being.

[31] Zhou X., Snoswell C.L., Harding L.E., Bambling M., Edirippulige S., Bai X., Smith A.C. (2020). The Role of Telehealth in Reducing the Mental Health Burden from COVID-19. Telemed. J. E Health.

[32] Luxton D.D., Pruitt L.D., Osenbach J.E. (2014). Best practices for remote psychological assessment via telehealth technologies. Prof. Psychol. Res. Pract..

[33] Riva G. (2000). From telehealth to E-health: Internet and distributed virtual reality in health care. Cyberpsychology Behav..

[34] Langarizadeh M., Tabatabaei M.S., Tavakol K., Naghipour M., Rostami A., Moghbeli F. (2017). Telemental health care, an effective alternative to conventional mental care: A systematic review. Acta Inform. Medica.

[35] Connolly S.L., Stolzmann K.L., Heyworth L., Weaver K.R., Bauer M.S., Miller C.J. (2021). Rapid Increase in Telemental Health within the Department of Veterans Affairs during the COVID-19 Pandemic. Telemed. e-Health.

[36] Madigan S., Racine N., Cooke J.E., Korczak D.J. (2021). COVID-19 and telemental health: Benefits, challenges, and future directions. Can. Psychol. Can..

[37] Wright J.H., Caudill R. (2020). Remote treatment delivery in response to the COVID-19 Pandemic. Psychother. Psychosom..

[38] Mishkind M.C., Shore J.H., Bishop K., D’Amato K., Brame A., Thomas M., Schneck C.D. (2020). Rapid Conversion to Telemental Health Services in Response to COVID-19: Experiences of Two Outpatient Mental Health Clinics. Telemed. e-Health.

[39] Luxton D.D., McCann R.A., Bush N.E., Mishkind M.C., Reger G.M. (2011). MHealth for mental health: Integrating smartphone technology in behavioral healthcare. Prof. Psychol. Res. Pract..

[40] Riva G., Wiederhold B.K., Mantovani F. (2019). Neuroscience of Virtual Reality: From Virtual Exposure to Embodied Medicine. Cyberpsychol. Behav. Soc. Netw..

[41] Valmaggia L.R., Latif L., Kempton M.J., Rus-Calafell M. (2016). Virtual reality in the psychological treatment for mental health problems: An systematic review of recent evidence. Psychiatry Res..

[42] Villani D., Grassi A., Cognetta C., Toniolo D., Cipresso P., Riva G. (2013). Self-help stress management training through mobile phones: An experience with oncology nurses. Psychol. Serv..

[43] Beatty L., Lambert S. (2013). A systematic review of internet-based self-help therapeutic interventions to improve distress and disease-control among adults with chronic health conditions. Clin. Psychol. Rev..

[44] Karyotaki E., van Ballegooijen W. (2020). Digital self-help interventions for suicidal ideation and behaviour. Lancet Digit. Heal..

[45] Yim S.H., Schmidt U. (2019). Experiences of computer-based and conventional self-help interventions for eating disorders: A systematic review and meta-synthesis of qualitative research. Int. J. Eat. Disord..

[46] Riva G., Wiederhold B.K. (2020). How Cyberpsychology and Virtual Reality Can Help Us to Overcome the Psychological Burden of Coronavirus. Cyberpsychol. Behav. Soc. Netw..

[47] Riva G., Mantovani F., Wiederhold B.K. (2020). Positive technology and COVID-19. Cyberpsychol. Behav. Soc. Netw..

[48] Pincus L.E. (2020). Telemental Health During a Global Pandemic: Clinical Lessons from Guided Self-Help, Telephone Therapy Case Studies. Pragmatic Case Stud. Psychother..

[49] Riva G., Riva E. (2020). COVID Feel Good: A Free VR Self-Help Solution for Providing Stress Management and Social Support During the COVID-19 Pandemic. Cyberpsychol. Behav. Soc. Netw..

[50] Fischer R., Bortolini T., Karl J.A., Zilberberg M., Robinson K., Rabelo A., Gemal L., Wegerhoff D., Nguyễn T.B.T., Irving B. (2020). Rapid Review and Meta-Meta-Analysis of Self-Guided Interventions to Address Anxiety, Depression, and Stress During COVID-19 Social Distancing. Front. Psychol..

[51] Bohil C.J., Alicea B., Biocca F.A. (2011). Virtual reality in neuroscience research and therapy. Nat. Rev. Neurosci..

[52] Salisbury J.P., Aronson T.M., Simon T.J. (2020). At-home self-administration of an immersive virtual reality therapeutic game for post-stroke upper limb rehabilitation. Proceedings of the CHI PLAY 2020—Extended Abstracts of the 2020 Annual Symposium on Computer-Human Interaction in Play.

[53] Donker T., van Klaveren C., Cornelisz I., Kok R.N., van Gelder J.-L. (2020). Analysis of Usage Data from a Self-Guided App-Based Virtual Reality Cognitive Behavior Therapy for Acrophobia: A Randomized Controlled Trial. J. Clin. Med..

[54] Riva G., Bernardelli L., Browning M.H.E.M., Castelnuovo G., Cavedoni S., Chirico A., Cipresso P., de Paula D.M.B., Di Lernia D., Fernández-Álvarez J. (2020). COVID Feel Good—An Easy Self-Help Virtual Reality Protocol to Overcome the Psychological Burden of Coronavirus. Front. Psychiatry.

[55] Italian Ministry of Health Covid-19. www.salute.gov.it/portale/news/p3_2_1_1_1.jsp?lingua=italiano&menu=notizie&p=dalministero&id=4186.

[56] Chirico A., Clewis R.R., Yaden D.B., Gaggioli A. (2021). Nature versus art as elicitors of the sublime: A virtual reality study. PLoS ONE.

[57] Gilbert P. (2009). Developing a compassion-focused approach in cognitive behavioural therapy. Cognitive Behaviour Therapy: A Guide for the Practising Clinician.

[58] Gilbert P. (2010). An introduction to Compassion Focused Therapy in Cognitive Behavior Therapy. Int. J. Cogn. Ther..

[59] Winch G. (2013). Emotional First Aid: Practical Strategies for Treating Failure, Rejection, Guilt, and Other Everyday Psychological Injuries.

[60] Lovibond P.F., Lovibond S.H. (1995). The structure of negative emotional states: Comparison of the Depression Anxiety Stress Scales (DASS) with the Beck Depression and Anxiety Inventories. Behav. Res. Ther..

[61] Bottesi G., Ghisi M., Altoè G., Conforti E., Melli G., Sica C. (2015). The Italian version of the Depression Anxiety Stress Scales-21: Factor structure and psychometric properties on community and clinical samples. Compr. Psychiatry.

[62] Cohen S., Kamarck T., Mermelstein R. (1983). Perceived Stress Scale.

[63] Mondo M., Sechi C., Cabras C. (2021). Psychometric evaluation of three versions of the Italian Perceived Stress Scale. Curr. Psychol..

[64] Beck A.T., Weissman A., Lester D., Trexler L. (1974). The measurement of pessimism: The Hopelessness Scale. J. Consult. Clin. Psychol..

[65] Innamorati M., Lester D., Balsamo M., Erbuto D., Ricci F., Amore M., Girardi P., Pompili M. (2014). Factor validity of the beck hopelessness scale in Italian medical patients. J. Psychopathol. Behav. Assess..

[66] Capanna C., Stratta P., Collazzoni A., D’Ubaldo V., Pacifico R., Di Emidio G., Ragusa M., Rossi A. (2013). Social connectedness as resource of resilience: Italian validation of the social connectedness scale—Revised. J. Psychopathol..

[67] Lee R.M., Robbins S.B. (1995). Measuring Belongingness: The Social Connectedness and the Social Assurance Scales. J. Couns. Psychol..

[68] Cosentino T., Pellegrini V., Giacomantonio M., Saliani A.M., Basile B., Saettoni M., Gragnani A., Buonanno C., Mancini F. (2020). Validation and psychometric properties of the Italian version of the Fear of Guilt Scale. Rass. Psicol..

[69] Ahorsu D.K., Lin C.Y., Imani V., Saffari M., Griffiths M.D., Pakpour A.H. (2020). The Fear of COVID-19 Scale: Development and Initial Validation. Int. J. Ment. Health Addict..

[70] Pedrabissi L., Santinello M. (1989). Verifica della validità dello S.T.A.I. forma Y di Spielberger. Boll. Psicol. Appl..

[71] Set S. (2010). State-Trait Anxiety Inventory for Adults. Garden.

[72] Smith J. (2000). Relaxation Inventory.

[73] Ruden R.A. Subjective Units of Distress Scale. http://haveningblog.tumblr.com/post/100433877079/suds-subjective-units-of-distress-scale.

[74] Cohen J. (2013). Statistical Power Analysis for the Behavioral Sciences.

[75] Heber E., Ebert D.D., Lehr D., Cuijpers P., Berking M., Nobis S., Riper H. (2017). The benefit of web-and computer-based interventions for stress: A systematic review and meta-analysis. J. Med. Internet Res..

[76] Luke S.G. (2017). Evaluating significance in linear mixed-effects models in R. Behav. Res. Methods.

[77] Bates D., Mächler M., Bolker B.M., Walker S.C. (2015). Fitting linear mixed-effects models using lme4. J. Stat. Softw..

[78] Bolker B.M. (2008). Ecological Models and Data in R.

[79] Kuznetsova A., Brockhoff P.B., Christensen R.H.B. (2017). lmerTest Package: Tests in Linear Mixed Effects Models. J. Stat. Softw..

[80] Lenth R., Lenth M.R. (2018). Package ‘lsmeans'. Am. Stat..

[81] Evans C., Margison F., Barkham M. (1998). The contribution of reliable and clinically significant change methods to evidence-based mental health. Evid. Based. Ment. Health.

[82] Morris S.B. (2008). Estimating effect sizes from pretest-posttest-control group designs. Organ. Res. methods.

[83] Page P. (2014). Beyond statistical significance: Clinical interpretation of rehabilitation research literature. Int. J. Sports Phys. Ther..

[84] Necho M., Tsehay M., Birkie M., Biset G., Tadesse E. (2021). Prevalence of anxiety, depression, and psychological distress among the general population during the COVID-19 pandemic: A systematic review and meta-analysis. Int. J. Soc. Psychiatry.

[85] Cipolletta S., Ortu M.C. (2020). COVID-19: Common constructions of the pandemic and their implications. J. Constr. Psychol..

[86] Wahlund T., Mataix-Cols D., Lauri K.O., de Schipper E., Ljótsson B., Aspvall K., Andersson E. (2021). Brief online cognitive behavioural intervention for dysfunctional worry related to the COVID-19 pandemic: A randomised controlled trial. Psychother. Psychosom..

[87] Piltch-Loeb R., Merdjanoff A., Meltzer G. (2021). Anticipated mental health consequences of COVID-19 in a nationally-representative sample: Context, coverage, and economic consequences. Prev. Med..

[88] Livingston N.A., Shingleton R., Heilman M.E., Brief D. (2019). Self-help smartphone applications for alcohol use, PTSD, anxiety, and depression: Addressing the new research-practice gap. J. Technol. Behav. Sci..

[89] Poletti B., Tagini S., Brugnera A., Parolin L., Pievani L., Ferrucci R., Compare A., Silani V. (2020). Telepsychotherapy: A leaflet for psychotherapists in the age of COVID-19. A review of the evidence. Couns. Psychol. Q..

[90] COVID Feel Good. https://www.covidfeelgood.com/.

