# Supplementary material for: A Virtual Reality-Based Self-Help Intervention for Dealing with the Psychological Distress Associated with the COVID-19 Lockdown: An Effectiveness Study with a Two-Week Follow-Up
Source: Int J Environ Res Public Health. 2021 Aug 2;18(15):8188. doi: 10.3390/ijerph18158188 (PMC8346162; doi:10.3390/ijerph18158188)
Supplement: Supplementary file 1 [file ijerph-18-08188-s001.zip › ijerph-1288212-supplementary.pdf]

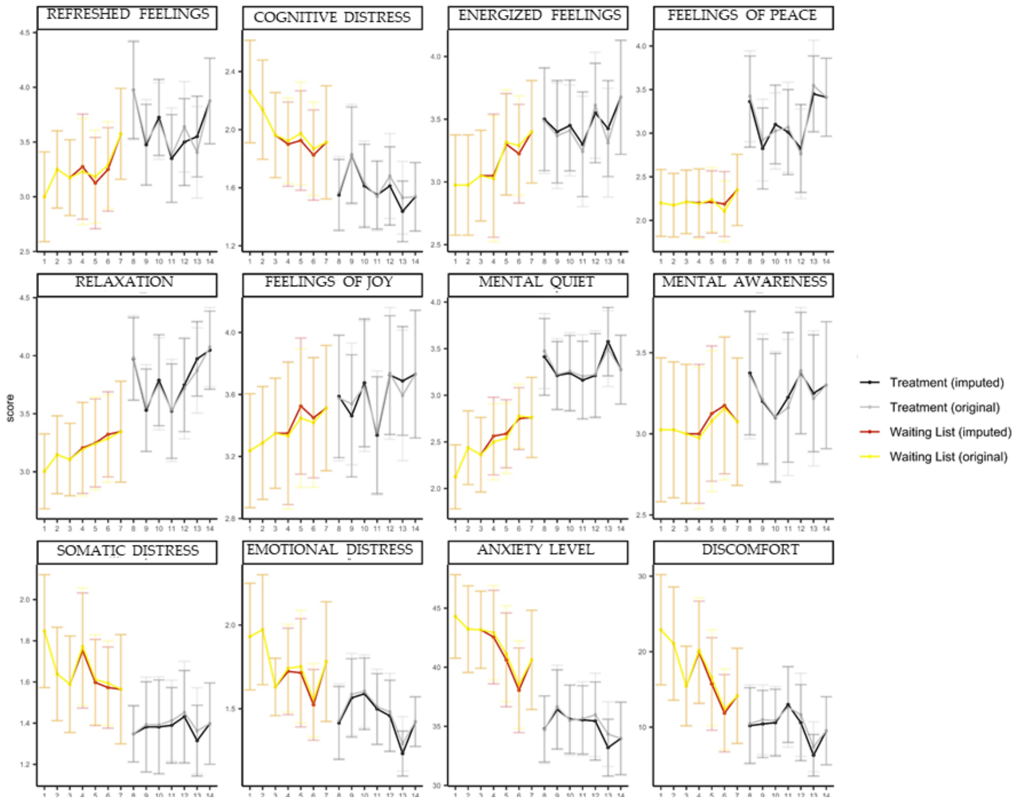

Figure S1. Results of imputation with random forest algorithm compared to real values. Daily changes in the subjective experience (refreshed feelings, SRSI3- Rest/Refresh; energized feelings; SRSI3- Energized, relaxation SRSI3 - Physical relaxation; feelings of peace, SRSI3- At ease/peace; feelings of joy, SRSI3- Joy; mental quiet, SRSI3- Mental quiet; mental awareness, SRSI3- Aware; somatic stress, SRSI3- Somatic stress; emotional stress, SRSI3- Emotional stress; cognitive stress, SRSI3- Cognitive stress; anxiety level, State-Trait Anxiety Inventory- State - STAI-S; discomfort,

Subjective Units of Distress Scale SUDS). State measures collected each day during the waiting week (7 days before the start of the intervention) and during the intervention phase, after each treatment module. Mean and 95% CI are depicted in the graphs.

Table S1. Estimated parameters for Linear Mixed Models fitted for primary outcome measures. For all variables, the first model (model 1) was run according to the formula= Outcome ~= ~1 | ID; the second model (model 2) was run according to the following formula = Outcome ~ Time= ~1 | ID, and the third model (model 3) according to this formula = Outcome ~ Time*Modality = ~1 | ID.

**Variables**		AIC	BIC	logLik	deviance	Chisq	Pr(>Chisq)
Depression Symptoms	Model 1	996.33	1005.5	-495.16	990.33		
	Model 2	983.39	1001.8	-485.69	971.39	18.9405	0.0002813
	Model 3	989.22	1019.8	-484.61	969.22	2.169	0.7045820
Anxiety Symptoms	Model 1	871.49	880.67	-432.74	865.49		
	Model 2	870.74	889.12	-429.37	858.74	6.7425	0.08057
	Model 3	873.71	904.33	-426.85	853.71	5.0383	0.28340
Stress Symptoms	Model 1	1060.8	1070.0	-527.42	1054.8		
	Model 2	1048.8	1067.2	-518.40	1036.8	18.0521	0.0004291
	Model 3	1051.7	1082.3	-515.83	1031.7	5.1449	0.2727492
General distress	Model 1	1068.3	1077.5	-531.14	1062.3		
	Model 2	1054.6	1073.0	-521.29	1042.6	19.6908	0.0001967
	Model 3	1057.7	1088.3	-518.83	1037.7	4.9248	0.2950959
Perceived stress level (	Model 1	973.02	982.21	-483.51	967.02		
	Model 2	964.16	982.54	-476.08	952.16	14.8570	0.001943
	Model 3	966.58	997.20	-473.29	946.58	5.5871	0.232182
Perceived hopelessness	Model 1	685.89	695.07	-339.94	679.89		
	Model 2	677.97	696.34	-332.98	665.97	13.9168	0.003021
	Model 3	684.88	715.51	-332.44	664.88	1.0851	0.896630
Perceived social connectedness	Model 1	1004.06	1013.2	-499.03	998.06		
	Model 2	994.32	1012.7	-491.16	982.32	15.744	0.001279

	Model 3	997.32	1028.0	-488.66	977.32	4.994	0.287913
Fear of Coronavirus	Model 1	819.49	828.68	-406.75	813.49		
	Model 2	810.96	829.34	-399.48	798.96	14.5296	0.002266
	Model 3	818.77	849.40	-399.39	798.77	0.1933	0.995621

Table S2. Estimated parameters for Linear Mixed Models fitted for state outcome measures. For all variables, the first model (model 1) was run according to the formula= Outcome ~= ~1 | ID; the second model (model 2) was run according to the following formula = Outcome ~ Time= ~1 | ID, and the third model (model 3) according to this formula = Outcome ~ Time*Immersion = ~1 | ID.

**Variables**		AIC	BIC	logLik	deviance	Chisq	Pr(>Chisq)
Anxiety level	Model 1	576.07	583.22	-285.04	570.07		
	Model 2	549.64	559.17	-270.82	541.64	28.4353	9.688e-08
	Model 3	552.68	566.97	-270.34	540.68	0.9573	0.6196
Refreshed feelings	Model 1	196.30	203.44	-95.148	190.30		
	Model 2	190.67	200.19	-91.333	182.67	7.6300	0.00574
	Model 3	189.86	204.15	-88.930	177.86	4.8064	0.09043
Energized feelings	Model 1	202.68	209.82	-98.338	196.68		
	Model 2	199.35	208.88	-95.674	191.35	5.3280	0.02099
	Model 3	200.86	215.15	-94.428	188.86	2.4923	0.28760
Perceived physical relaxation	Model 1	255.62	262.76	-124.81	249.62		
	Model 2	237.20	246.72	-114.60	229.20	20.4223	6.21e-06
	Model 3	240.81	255.10	-114.41	228.81	0.3837	0.8254
Feelings of peace	Model 1	420.46	427.60	-207.23	414.46		
	Model 2	417.42	426.95	-204.71	409.42	5.0330	0.02487
	Model 3	421.29	435.58	-204.65	409.29	0.1317	0.93627
Feelings of joy	Model 1	199.06	206.20	-96.528	193.06		
	Model 2	197.28	206.81	-94.642	189.28	3.7717	0.05213
	Model 3	199.41	213.70	-93.706	187.41	1.8721	0.39217
Feelings of mental quiet	Model 1	225.20	232.34	-109.599	219.20		

	Model 2	203.28	212.81	-97.643	195.28	23.9131	1.008e-06
	Model 3	206.55	220.84	-97.274	194.55	0.7364	0.692
Feelings of mental awareness	Model 1	220.02	227.17	-107.01	214.02		
	Model 2	220.94	230.46	-106.47	212.94	1.0850	0.2976
	Model 3	223.94	238.23	-105.97	211.94	0.9988	0.6069
Perceived somatic distress	Model 1	98.277	105.423	-46.139	92.277		
	Model 2	76.449	85.977	-34.224	68.449	23.8283	1.053e-06
	Model 3	78.983	93.275	-33.492	66.983	1.4655	0.4806
Perceived emotional distress	Model 1	129.80	136.94	-61.899	123.80		
	Model 2	118.55	128.08	-55.275	110.55	13.2477	0.0002729
	Model 3	122.52	136.82	-55.262	110.52	0.0262	0.9869642
Perceived cognitive stress	Model 1	161.83	168.97	-77.915	155.83		
	Model 2	140.18	149.70	-66.088	132.18	23.653	1.154e-06
	Model 3	143.78	158.07	-65.889	131.78	0.399	0.8192
Subjectively perceived levels of discomfort	Model 1	634.96	642.11	-314.48	628.96		
	Model 2	616.85	626.37	-304.42	608.85	20.1127	7.301e-06
	Model 3	618.43	632.72	-303.21	606.43	2.4168	0.2987

Table S3. Bonferroni adjusted-pairwise comparisons for all primary and secondary outcome measure across the different time points. (7 days before the start of the intervention – Waiting Period, before the start of the intervention, Day 0 – T0; end of the intervention, Day 7 – T1; 2-week follow-up, Day 21 – T2).

Constrast	estimate	SE	df	t.ratio	p.value	d	CI	CI_low	CI_high	Variables
WaitingList - TimeT0	-0.10	0.90	121.07	-0.11	1	-0.01	0.95	-0.18	0.16	Depression Symptoms
WaitingList – TimeT1	3.10	0.90	121.07	3.41	**0.005**	0.31	0.95	0.12	0.49	Depression Symptoms
WaitingList – TimeT2	2.52	0.92	121.38	2.72	**0.043**	0.24	0.95	0.06	0.42	Depression Symptoms
TimeT0 – TimetT1	3.20	0.90	121.07	3.52	**0.003**	0.32	0.95	0.13	0.50	Depression Symptoms
Time0 – TimeT2	2.62	0.92	121.38	2.83	**0.032**	0.25	0.95	0.07	0.43	Depression Symptoms
TimeT1 – TimeT2	-0.58	0.92	121.38	-0.62	1	-0.05	0.95	-0.23	0.12	Depression Symptoms
WaitingList - TimeT0	-0.85	0.66	115	-1.28	1	-0.11	0.95	-0.30	0.06	Anxiety Symptoms
WaitingList – TimeT1	0.85	0.66	115	1.28	1	0.11	0.95	-0.06	0.30	Anxiety Symptoms
WaitingList – TimeT2	0.25	0.67	115.40	0.37	1	0.03	0.95	-0.14	0.21	Anxiety Symptoms
TimeT0 – TimeT1	1.70	0.66	115	2.56	0.07	0.23	0.95	0.05	0.42	Anxiety Symptoms
TimeT0 - TimeT2	1.10	0.67	115.401	1.63	0.62	0.15	0.95	-0.03	0.33	Anxiety Symptoms
TimeT1 – TimeT2	-0.60	0.67	115.40	-0.88	1	-0.08	0.95	-0.26	0.10	Anxiety Symptoms
WaitingList - TimeT0	-0.35	1.13	115	-0.30	1	-0.02	0.95	-0.21	0.15	Stress Symptoms
WaitingList – TimeT1	3.70	1.13	115	3.25	**0.008**	0.30	0.95	0.11	0.48	Stress Symptoms
WaitingList – TimeT2	2.88	1.15	115.33	2.49	0.08	0.23	0.95	0.04	0.41	Stress Symptoms
TimeT0 – TimeT1	4.05	1.13	115	3.56	**0.003**	0.33	0.95	0.14	0.51	Stress Symptoms
TimeT0 – TimeT2	3.23	1.15	115.33	2.79	**0.036**	0.26	0.95	0.07	0.44	Stress Symptoms
TimeT1 – TimeT2	-0.82	1.15	115.33	-0.71	1	-0.06	0.95	-0.24	0.11	Stress Symptoms
WaitingList - TimeT0	-0.65	1.14	115	-0.56	1	-0.05	0.95	-0.23	0.12	General distress

WaitingList – TimeT1	3.82	1.14	115	3.35	**0.006**	0.31	0.95	0.12	0.49	General distress
WaitingList – TimeT2	2.70	1.16	115.29	2.32	0.12	0.21	0.95	0.03	0.40	General distress
TimeT0 – TimeT1	4.47	1.14	115	3.92	**> 0.001**	0.36	0.95	0.17	0.55	General distress
TimeT0 – TimeT2	3.35	1.16	115.29	2.88	**0.027**	0.26	0.95	0.08	0.45	General distress
Time7 – TimeT2	-1.12	1.16	115.29	-0.96	1	-0.09	0.95	-0.27	0.09	General distress
WaitingList - TimeT0	-0.47	0.86	115	-0.54	1	-0.05	0.95	-0.23	0.13	Perceived stress level
WaitingList – TimeT1	2.22	0.86	115	2.56	0.07	0.23	0.95	0.05	0.42	Perceived stress level
WaitingList – TimeT2	2.11	0.88	115.33	2.39	0.11	0.22	0.95	0.03	0.40	Perceived stress level
TimeT0 - Time7	2.70	0.86	115	3.10	**0.014**	0.28	0.95	0.09	0.47	Perceived stress level
TimeT0 – TimeT2	2.59	0.88	115.33	2.93	**0.024**	0.27	0.95	0.08	0.45	Perceived stress level
TimeT1- TimeT2	-0.11	0.88	115.33	-0.12	1	-0.01	0.95	-0.19	0.17	Perceived stress level
WaitingList - TimeT0	-0.02	0.94	115	-0.02	1	-0.00	0.95	-0.18	0.18	Perceived social connectedness
WaitingList – TimeT1	-2.70	0.94	115	-2.85	**0.030**	-0.26	0.95	-0.45	-0.08	Perceived social connectedness
WaitingList – TimeT2	-2.78	0.96	115.30	-2.89	**0.026**	-0.26	0.95	-0.45	-0.08	Perceived social connectedness
TimeT0 – TimeT1	-2.67	0.94	115	-2.83	**0.032**	-0.26	0.95	-0.44	-0.07	Perceived social connectedness
Time0 – TimeT2	-2.76	0.96	115.30	-2.87	**0.029**	-0.26	0.95	-0.45	-0.08	Perceived social connectedness
TimeT1 – TimeT2	-0.08	0.96	115.30	-0.08	1	-0.00	0.95	-0.19	0.17	Perceived social connectedness
WaitingList - TimeT0	0.40	0.31	115	1.27	1	0.11	0.95	-0.06	0.30	Perceived hopelessness
WaitingList – TimeT1	1.15	0.31	115	3.67	**0.002**	0.34	0.95	0.15	0.52	Perceived hopelessness
WaitingList – TimeT2	0.33	0.31	115.14	1.03	1	0.09	0.95	-0.08	0.27	Perceived hopelessness
TimeT0 – TimeT1	0.75	0.31	115	2.39	0.10	0.22	0.95	0.03	0.40	Perceived hopelessness
Time0 - Time21	-0.07	0.31	115.14	-0.22	1	-0.02	0.95	-0.20	0.16	Perceived hopelessness
Time7 - Time21	-0.82	0.31	115.14	-2.57	0.06	-0.23	0.95	-0.42	-0.05	Perceived hopelessness

WaitingList - Time0	0.80	0.47	115	1.68	0.57	0.15	0.95	-0.02	0.34	Fear of Coronavirus
WaitingList - Time7	1.67	0.47	115	3.51	**0.003**	0.32	0.95	0.13	0.51	Fear of Coronavirus
WaitingList - Time21	1.48	0.48	115.13	3.05	**0.016**	0.28	0.95	0.09	0.47	Fear of Coronavirus
Time0 - Time7	875	0.47	115	1.83	0.41	0.17	0.95	-0.01	0.35	Fear of Coronavirus
Time0 - Time21	0.68	0.48	115.13	1.39	0.98	0.13	0.95	-0.05	0.31	Fear of Coronavirus
Time7 - Time21	-0.20	0.48	115.13	-0.40	1	-0.03	0.95	-0.22	0.14	Fear of Coronavirus
